# Applying the exposome concept to working life health: The EU EPHOR project

**DOI:** 10.1097/EE9.0000000000000185

**Published:** 2022-02-17

**Authors:** Anjoeka Pronk, Miranda Loh, Eelco Kuijpers, Maria Albin, Jenny Selander, Lode Godderis, Manosij Ghosh, Roel Vermeulen, Susan Peters, Ingrid Sivesind Mehlum, Michelle C. Turner, Vivi Schlünssen, Marcel Goldberg, Manolis Kogevinas, Barbara N. Harding, Svetlana Solovieva, Tina Garani-Papadatos, Martie van Tongeren, The EPHOR Consortium, Rob Stierum

**Affiliations:** a Nederlandse Organisatie voor Toegepast Natuurwetenschappelijk Onderzoek, Utrecht, the Netherlands; b Institute of Occupational Medicine, Edinburgh, United Kingdom; c Institute of Environmental Medicine, Unit of Occupational Medicine, Karolinska Institute, Stockholm, Sweden; d Environment and Health, Department of Public Health and Primary Care, KU Leuven, Leuven, Belgium; e Institute for Risk Assessment Sciences, Utrecht University, Utrecht, the Netherlands; f National Institute of Occupational Health (STAMI), Oslo, Norway; g Barcelona Institute for Global Health (ISGlobal), Barcelona, Spain; h Department of Public Health, Section of Environment, Occupation and Health, Danish Ramazzini Centre, Aarhus University, Aarhus, Denmark; i Inserm, Population-Based Epidemiological Cohorts Unit, France; j Finnish Institute of Occupational Health, University of Helsinki, Helsinki, Finland; k School of Public Health, University of West Attica, Athens, Greece; l Centre for Occupational and Environmental Health, The University of Manchester, Manchester, United Kingdom.

**Keywords:** Exposome, Working life, Occupational, Noncommunicable disease

## Abstract

Exposures at work have a major impact on noncommunicable diseases (NCDs). Current risk reduction policies and strategies are informed by existing scientific evidence, which is limited due to the challenges of studying the complex relationship between exposure at work and outside work and health. We define the working life exposome as all occupational and related nonoccupational exposures. The latter includes nonoccupational exposures that may be directly or indirectly influenced by or interact with the working life of the individual in their relation to health. The Exposome Project for Health and Occupational Research aims to advance knowledge on the complex working life exposures in relation to disease beyond the single high exposure–single health outcome paradigm, mapping and relating interrelated exposures to inherent biological pathways, key body functions, and health. This will be achieved by combining (1) large-scale harmonization and pooling of existing European cohorts systematically looking at multiple exposures and diseases, with (2) the collection of new high-resolution external and internal exposure data. Methods and tools to characterize the working life exposome will be developed and applied, including sensors, wearables, a harmonized job exposure matrix (EuroJEM), noninvasive biomonitoring, omics, data mining, and (bio)statistics. The toolbox of developed methods and knowledge will be made available to policy makers, occupational health practitioners, and scientists. Advanced knowledge on working life exposures in relation to NCDs will serve as a basis for evidence-based and cost-effective preventive policies and actions. The toolbox will also enable future scientists to further expand the working life exposome knowledge base.

**What this study adds**

The European Union Exposome Project for Health and Occupational Research (EPHOR) will contribute uniquely to exposome research by characterizing the working life exposome as an essential factor in the development of noncommunicable diseases (NCDs). The results will serve as a basis for evidence-based and cost-effective preventive policies and actions, ultimately contributing to reducing the burden of NCDs. Also, the focus on occupational exposure settings, with well-defined populations and higher exposure levels that can generally be well characterized, may provide a unique setting for developing and demonstrating exposome methods. This article describes the objectives, approach, methods, expected outcomes, and expected impact of EPHOR.

## Introduction

Nearly all occupational diseases in high-income countries are noncommunicable diseases (NCDs), especially cancers, cardiovascular, respiratory, neurodegenerative, mental, and musculoskeletal diseases.^1–3^ External factors play an important role in the causation or exacerbation of NCDs, and various occupational exposures have been studied in relation to NCDs. Examples are particulate and chemical exposures (e.g., diesel, silica, or benzene) linked to respiratory disease and cancer, noise linked to hearing loss, and heavy lifting and vibrations linked to musculoskeletal disorders.^4^ Conservative estimates of the global burden of occupational disease vary between 3% and 7% of global mortality, translating to 1.5 to 2.3 million deaths each year.^5–7^ In the European Union (EU), approximately 300,000 work-related deaths per year are estimated resulting in economic losses of around 3% to 4% of gross domestic product.^3,8^ In its Strategic Framework on Health and Safety at work 2021 to 2027,^8^ the EU emphasizes the need for improving prevention of workplace illnesses in line with its “Vision Zero” vision, aimed at eliminating work-related deaths and reducing work-related illnesses by 2030. Ensuring a safe and healthy work environment is also a strategic goal for many national governments and of critical importance for both employers and employees.

Current risk reduction policies and strategies are informed by existing scientific evidence on the burden of occupational NCDs. However, today’s knowledge of exposure-disease associations is incomplete. First, it is limited to a specific set of known risk factors typically studied based on a “one exposure, one disease” approach in occupational settings with a high single occupational exposure in relation to a common health end point. For instance, the Global Burden of Disease project has based its burden of occupational disease estimate on exposure-disease associations for a range of single risk factors and NCDs, i.e., 14 different carcinogens for 7 types of cancer, asthmagens for asthma, particulate matter, gases, and fumes for chronic obstructive pulmonary disease, noise for hearing loss, and ergonomic risk factors for low back pain.^9^

These associations likely only represent the tip of the iceberg given that exposure-disease associations also involve lower level exposures and are mainly multifactorial. Further, working and lifestyle are increasingly intertwined and a persons’ employment may also affect general exposures and lifestyle. Secondly, knowledge regarding vulnerable groups is limited. For more efficient prevention of disease, more diversity-sensitive risk characterization is needed with regard to, e.g., age and gender.^10^ The working population over 55 years of age in the EU is expected to increase by at least 16% between 2010 and 2030.^11^ An aging workforce may be at increased risk for developing NCDs due to concomitant age-related diseases or may have reduced resilience to accumulated risk factors.^12^ In addition, although over the last decades female participation in the workforce has risen, work-related risks to women’s health are less studied.^13^ For instance, even studies of occupational risks for pregnant women have focused on fetal effects for decades, but recent studies also suggest an increased risk of pregnancy-related health effects in women exposed to physical risk factors like noise and vibration.^14,15^ Lastly, insights into informative biological pathways and biomarkers to link exposure to health are mostly lacking. These could contribute to the quantitative understanding of exposure-response associations and provide agnostic discovery of biomarkers for exposure monitoring.

New approaches are needed to address these knowledge gaps. The need for new approaches is also emphasized by the nature of work, which is changing in many countries. Smaller companies in terms of workforce, increased frequency of job changes, and more migrating workers are resulting in more heterogeneous work patterns and work forces. This complicates occupational exposure assessment and poses challenges to the power of studies relating these to health outcomes, until now typically based on groups of workers with homogeneous exposures throughout their entire working lifetime.

The exposome, which encompasses all nongenetic risk factors experienced during a person’s life (external exposome) and its relation to biological responses inside the human body (internal exposome), is a promising concept for exploring the complex relationships between environment and disease.^16^ An exposome approach is better suited for unraveling complex exposure patterns in relation to disease, beyond the single high exposure–single health outcome paradigm, to mapping and linking interrelated exposures to inherent biological pathways, key body functions, and health, offering a more holistic approach for investigating how the working life environment may cause NCDs. The exposome approach is more focused on individuals or smaller exposure groups and will be better suited for identification of vulnerable subgroups, as well as for studying occupational exposures in the “new world of work” compared to traditional exposure and risk assessment approaches.

**Figure 1.**
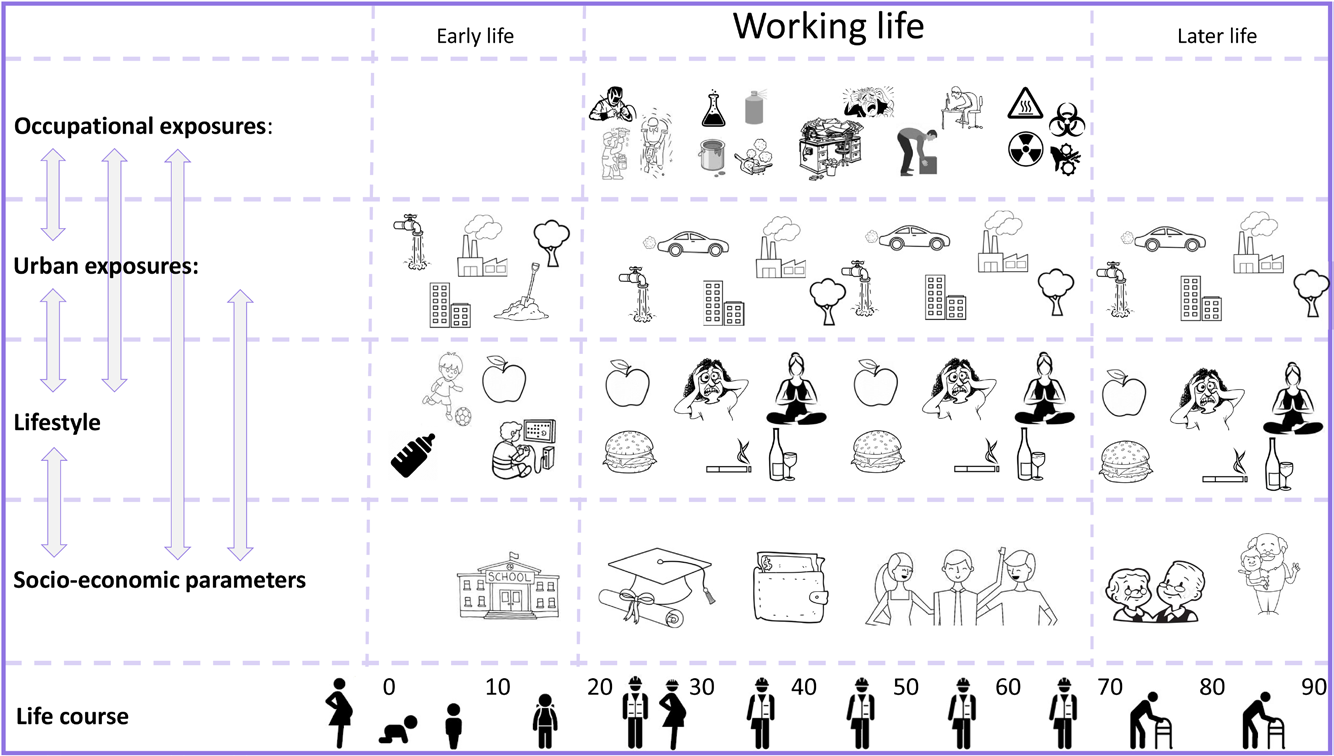
Working life exposures in the exposome context.

Until now, work-related exposures have been largely neglected in exposome studies, despite the fact that the working life makes up a major part of the total lifespan including important vulnerable life stages, work-related exposures are typically higher and more frequent than urban exposures, and occupation closely relates to lifestyle, behaviour and socio-economic status (SES) (Figure 1).

In the Exposome Project for Health and Occupational Research (EPHOR), we define the working life exposome as the exposure to all occupational and related nonoccupational (e.g., urban, lifestyle, and SES) factors. This definition includes nonoccupational exposures that may be directly or indirectly influenced by or interact with the working life of an individual in their relation to health. For example during shift work, dietary habits and sleep patterns may differ significantly from nonshift work. Also, occupation is a determinant of SES, which may affect urban exposures, lifestyle, and stress. EPHOR will embrace the working life exposome paradigm in order to advance knowledge on the heterogeneous working life exposure patterns in relation to common NCDs, with focus on (1) uncovering the “hidden part of the iceberg” including effects of lower magnitude, diseases of lower prevalence, and more complex exposure interactions; (2) identification of vulnerable life stages and subgroups; (3) mechanistic insights, including identification of biomarkers of exposure and early disease; and (4) exposure-response associations for more short-term and higher resolution exposures. This article describes the objectives, approach, methods, expected outcomes, and impact of the EPHOR project.

## Project description

### Aim and objectives

The ultimate aim of EPHOR (https://www.ephor-project.eu/) is to apply the exposome concept to working life health research in order to improve the evidence base for developing cost-effective preventive actions, ultimately improving health and reducing the burden of NCDs. The objectives in EPHOR are to develop

- Better and more complete knowledge on how multiple exposures within the working life exposome are related to the occurrence of NCDs like cancers and cardiovascular, respiratory, musculoskeletal, mental, metabolic, and neurodegenerative diseases, including complex interactions of exposures, biological pathways and early signs of health damages, and vulnerability at different life stages;
- Innovative methods for collection, storage, and interpretation of working life exposome data, including the health, economic, and societal impact of interventions.

Both the developed knowledge and methods will be made available in a working life exposome toolbox to three stakeholder groups: health scientists, occupational health practitioners, and policy makers.

### Approach

**Figure 2.**
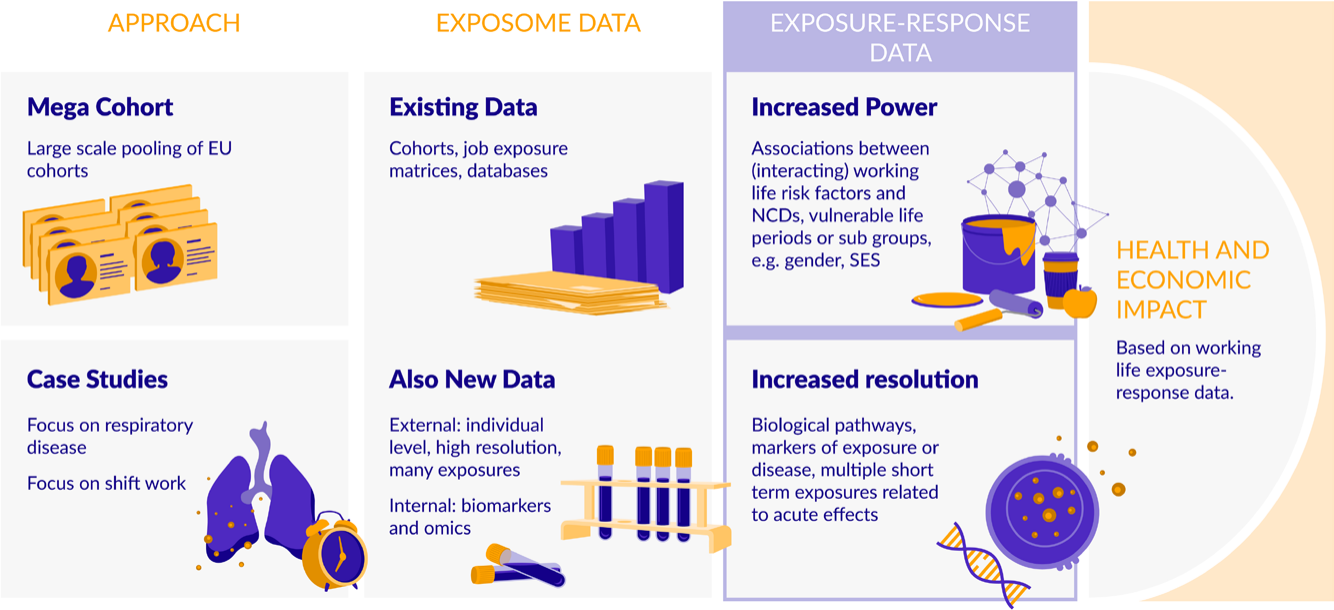
EPHOR approach combining the EPHOR mega cohort and focused case studies.

Two different study designs are combined in order to advance knowledge on the heterogeneous working life exposure patterns in relation to NCDs: (1) systematic and agnostic analyses of a wide range of NCDs in a pooled mega cohort making use of the large body of existing occupational cohorts and population-based cohorts across Europe; (2) detailed analyses in case studies making use of the advancing technologies for collecting internal and external exposome data. In addition, EPHOR will develop concepts and methods on how to apply these data to perform impact assessments (Figure 2).

Most of our current knowledge on occupational health is based on industry-based cohorts focusing on specific occupational situations or exposures or general population cohorts in which occupational exposures are considered along with documenting general life exposures. These cohorts are invaluable resources for obtaining a detailed understanding of the working life exposome in relation to health. However, these cohorts have limited power for exposome approaches focused on detecting effects of lower magnitude, disease of lower prevalence complex interactions, or vulnerable subgroups and life stages. By large-scale pooling of existing cohorts, the EPHOR Mega Cohort approach will achieve sufficient power to

- Move away from single high exposure, single common disease evaluations to the systematic and agnostic exposome-based exploration of combinations of risk factors in relation to NCDs, including rare exposures or lower level exposures to known risk factors and rarer diseases such as rare cancers (e.g., breast cancer in men, sarcomas), hemorrhagic stroke, or malformations at birth;
- Identify vulnerable life stages and population subgroups in which these risk factors result in more pronounced or different health effects.

These cohort studies rely primarily on external exposure estimates at the (job or industry) group level. In two case studies, we will take advantage of recent technological advances for the collection, storage and analyses of more (time resolved) individual-level external and internal exposure data that enable capturing the variety and dynamics of a multitude of exposures in order to

- Obtain mechanistic insights linking external exposure to early and long-term effects and from these identify early biomarkers indicative for disease development;
- Study exposure-response relations with individual-level data at a higher resolution.

### Study populations and design

**Figure 3.**
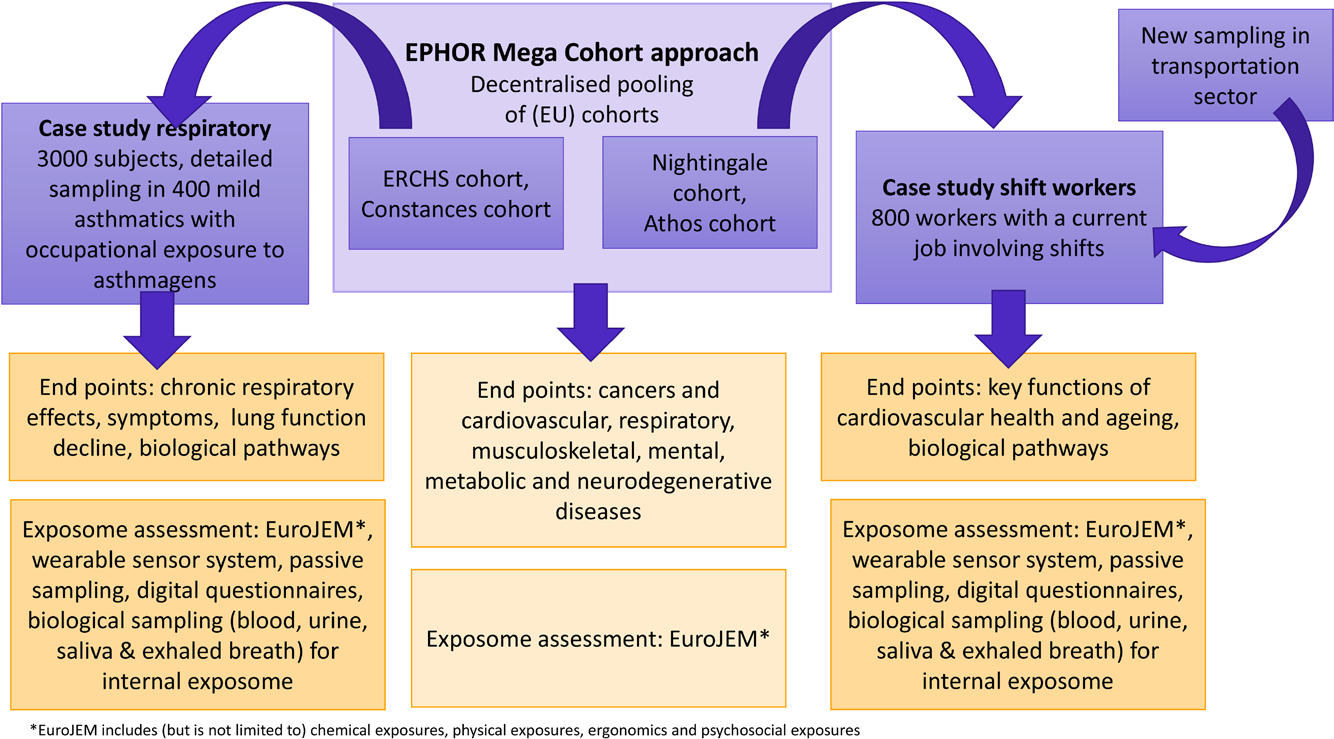
Study populations and design.

An overview of the EPHOR Mega Cohort approach and the case studies is given in Figure 3, along with the main end points studied and methods for assessing the exposome. A more detailed description is given below.

### EPHOR mega cohort approach

For the EPHOR Mega Cohort approach, we build on an inventory of European cohorts, with extensive information on employment history conducted in collaboration with the OMEGA-NET project.^17,18^ This inventory (which is available at: https://occupationalcohorts.net/) currently includes over 140 cohorts adding up to millions of subjects and continues to grow. Actual pooling of cohort data across country and institute boundaries is challenging due to privacy and ethics legislations. Therefore, a framework is being developed using DataSHIELD^19,20^ for joint decentralized analyses across cohorts, through virtual pooling of the individual cohorts. Meta-analysis will be used when virtual pooling is not possible. Through this framework, combinations of cohorts that are selected for each research question can be flexibly combined, resulting in a dynamic EPHOR Mega Cohort. A selected number of cohorts directly linked to the EPHOR consortium partners (≈10) has been invited for a pilot phase in which methods for data harmonization, meta data cataloging, and decentralized data analyses are developed. Additional cohorts will be invited for Mega Cohort analyses after research questions, and end points have been defined.

Data on health end points, relevant nonoccupational exposures that may be directly or indirectly linked to the working life, and potential confounders will be harmonized across the cohorts. A defined set of core variables that should be derivable by the majority of relevant cohorts and will be frequently used as covariates in working life research (e.g., employment history, smoking status, socioeconomic status, region/address, age) has been defined. A data harmonization protocol is being developed based on the available variable labels, categories, and values for these variables in the pilot phase cohorts. This harmonization protocol will guide local harmonization of data by cohort owners. Based on these harmonized core variables, an online data catalogue will be developed. Employment histories will be used to estimate occupational exposures by EuroJEM, explained in more detail below.

The following types of targeted research questions will be addressed in the Mega Cohort analyses: What are the most important working life risk factors for the development of a specific NCD over the life course? To what extent do working life exposures (including occupational, lifestyle, and SES) interact in their relation to an NCD? Are certain subpopulations more vulnerable? Can critical exposure time windows across the life course be identified? These targeted analyses will be performed in order to address predefined research questions focusing on specific (sets of) exposures and health outcomes. These research questions will be based on knowledge gaps identified in the occupational health literature that may be addressed by an exposome approach. The research questions will be prioritized based on availability of relevant cohorts and study power considerations (e.g., suspected prevalence of disease and exposures). For generating hypotheses on previously unknown or nonsuspected risk factors, a hierarchical modeling approach is being developed. This includes first assessing the association between job title and a specific NCD and in a second step add EuroJEM-based exposure factors to these models. For jobs for which EuroJEM does not explain the association between job and NCDs, probably unknown or nonsuspected exposures are responsible, laying the ground for new hypotheses.

### Case studies

#### Case study 1: respiratory health and the working life exposome

The following research questions will be addressed: How does long-term exposure to the working life exposome affect (prognosis of) chronic respiratory effects? Is this influenced by biomarkers of susceptibility, gender, or age? How does short-term exposure to the working life exposome affect lung function, respiratory symptoms, and effect biomarkers among asthmatics? Can key biological pathways and markers for exposure and respiratory health effects associated with the working life exposome be identified?

The study population derives from two population-based cohorts with (respiratory) health information, biological samples, and lifelong job histories with a planned follow-up in 2021: the European Community Respiratory Health Survey (ECRHS)^21^ and the French Constances Cohort.^22^ In total, 4000 subjects who participated in ECRHS between 2010 and 2012 or in Constances between 2012 and 2016 will be invited for the EPHOR-specific follow-up. A participation rate of 75% is expected, resulting in approximately 3000 individuals with EPHOR-specific follow-up data.

Follow-up among these individuals will include blood sampling, urine sampling, measurement of lung function, and questionnaires. Among these, a subgroup of around 400 mild asthmatics, with current occupational exposure to airborne irritants, will be selected. Jobs with airborne exposure to irritants will be identified based on a job exposure matrix (JEM).^23^ These 400 individuals will be subjected to a detailed one-week study, in which new data on external exposure, internal exposure, biomarkers, and omics will be collected, described below. Additionally, daily data on lung function and respiratory symptoms will be collected.

#### Case study 2: night shift work and the working life exposome

The following research questions will be addressed: How does the long-term and short-term working life exposome among shift workers affect key body functions of cardiovascular health and aging? Are there susceptibility factors influencing adverse effects of night shift? What are key biological pathways for health effects associated with shift?

The study will involve sampling in two types of populations. First, a newly recruited population of transportation workers in Spain is being recruited for whom shift work is common, including male and female workers with long-term rotating shifts. Second, we will make use of the Nightingale cohort in the Netherlands that includes both night and day shift female nurses^24^ (n = 59,947) and a cohort of Swedish health care employees^25^ (n = 60,000). In total, approximately 800 participants currently working in a job involving night shifts or day shifts will be recruited.

Among these individuals, new external exposure, internal exposure, biomarkers, and omics data will be collected, as described below for analyses of pathways related to shift work. The exposome protocol will also incorporate analyses to evaluate key body functions closely related to aging (e.g., metabolic syndrome, cognitive function, and hallmarks of aging^26^) and the development of cardiovascular disease through a combination of biomarkers, biochemical analyses, harmonized tests, clinical evaluations, and questionnaires.

### Methods for collection of working life exposome data

#### EuroJEM for large-scale harmonized exposure assessment in pooled cohorts

A JEM is a tool used to assess occupational external exposure to potential risk factors in large populations.^27^ By linking exposure estimates to job codes, job histories can be translated into specific exposures in a systematic and unbiased way. JEMs provide a standardized exposure assessment within and between studies with any misclassification expected to be nondifferential with respect to the health outcome.^28^ Several national JEMs have been developed in Europe^29–34^ for specific studies, populations, regions, and time periods, making use of different occupational coding systems and exposure definitions and classifications. This heterogeneity complicates the combined application of these individual JEMs at the European level in the EPHOR Mega Cohort analyses. In addition, several regions, populations, and time periods are not covered by existing JEMs, which cannot uncritically be extrapolated to other regions and time periods.

A tool for harmonized retrospective external working life exposure assessment across Europe and time periods will be developed to assess occupational exposure histories in the EPHOR Mega Cohort analyses and the case studies. This EuroJEM will include multiple relevant occupational and nonoccupational exposures, containing but not limited to chemical exposures (e.g., asbestos, quartz, chormium, wood dust), physical exposure (e.g., noise, ultra violet [UV] light), ergonomic exposures (e.g., heavy lifting, working with arms above shoulders), and psychosocial factors (job control, job strain, nonstandard working conditions). It is being constructed by combining and harmonizing existing JEMs. The EPHOR consortium has access to a large number of existing JEMs^30,35,36^ and will also include additional JEMs identified in the literature. To minimize exposure misclassification, the different JEMs will undergo job coding standardization and exposure assignments across different JEMs will be harmonized by job and exposure agent. For updating EuroJEM when new data occur, a protocol for including new working life exposure data into EuroJEM will be developed and tested, including methods for data mining and (Bayesian) decision criteria to determine if and how to revise exposure estimates. This will make EuroJEM dynamic, enhance transparency, and may lead to a higher granulation of exposure estimates within job titles (e.g., gender or industry specific). It can also aid the inclusion of new emerging risks in the future.

A major challenge for the utilization of any JEM is the process of translating job titles into meaningful exposure estimates. This requires free text fields from job histories to be coded using standardized occupational coding systems across studies (e.g., ISCO-68, ISCO-88). Therefore, improved methods for automated coding of free text fields in occupational histories will be developed based on artificial intelligence mimicking human experts for coding. Also, current job coding systems have been developed for economic purposes but often do not optimally reflect exposure categories. Improved coding systems will be developed and used for more flexible coding that allows for different coding structures depending on the risk factor, by expanding on existing descriptive clustering approaches to map job descriptions and estimated exposures using previous expert assessments of exposure.

#### New methods for high-resolution assessment of external exposure in the case studies

Conventional occupational exposure assessment mainly employs passive or active sampling with laboratory analyses for one or a few substances, resulting in time-weighted average (e.g., 8 hours) concentrations. Sampling and laboratory costs typically limit the number of samples. Wearable sensors, passive sampling, and smart technologies like ecological momentary assessments can potentially provide more temporal, spatial, and chemical resolution enabling enhanced assessment of an individual’s exposome.^37^ The following developments will be made and deployed in the two case studies.

#### Wearable sensor system

Besides collection of high-resolution data, low-cost sensors require less field researcher labor and related costs, while enabling the collection of a wider range of exposures by combining sensors into one system. This approach of longitudinal personal monitoring has been applied in short-term studies of air pollution health effects but thus far not in studies aimed at examining the role of occupational exposure in health. Types of low-cost sensors in exposome studies of the general environment have included temperature, UV, light, noise, several air pollutants including particulates, and location tracking.^38^ Their deployment for exposure assessment has been enabled by recent technical developments, such as the miniaturization of electronic components, the accessibility of low-cost computing processors, and the improved performance of electric batteries.^39^ However, challenges remain with respect to the reliability of low-cost sensors^38,40,41^ and interpretation and use of sensor data in exposome studies. Applying a system of multiple sensors throughout the day at work and away from work opens up prospects for addressing heterogeneous exposure patterns throughout a workday or for assessing exposure both at and outside the workplace. Within the occupational setting, the application of low-cost sensors for exposure assessment is limited to some pilot studies^42^ in industrial settings with sensors ranging from particulates, volatile organic compounds (VOCs), formaldehyde, hydrocarbons, and acids and nonchemical stressors like heat and noise. Within EPHOR, a wearable sensor system has been developed including sensors for fine dust, light, noise, UVB, physical activity, and sleep. The systems will be applied in (subsets of) both case studies to collect continuous exposure data during one week. Data gathered each day will be downloaded via a wireless gateway system, which can securely store data offline and upload data to a secure cloud server.

#### Passive sampling

Passive sampling methods allow for easier collection of larger amounts of exposure samples for laboratory analyses. Advances in analytical chemistry and informatics enable untargeted screening of a wide range of substances, allowing us to screen for potential unsuspected exposures that may be of interest and to understand the breadth of compounds workers may be exposed to in their daily lives. This approach moves beyond the single exposure paradigm, instead generating new hypotheses about exposure-disease relationships. The material often used for silicone wristband sampling^43^ (polydimethylsiloxane [PDMS]) has been coupled with a Tenax TA sampling tube to create a small wearable passive sampler for taking personal exposure samples during a week long period. The Tenax TA will be analyzed for volatile organic compounds, and the PDMS will be analyzed for semivolatile compounds.^43,44^ For the respiratory case study, electrostatic stationary dust samplers will be employed to collect dust samples in the participants’ home during one week and will be analyzed for microbial abundance and diversity using a microbiome approach.^45,46^

#### Digital questionnaires

To supplement sensor and passive sampler data collection, an app-based questionnaire^47^ will be used to obtain information on daily habits and symptoms. This system involves questions on daily sleep and wakening times, commute times, working times, food/drink consumption times, use of personal protective equipment, stress, other exposure factors, symptoms, and contextual information regarding the occupational setting, i.e., use of personal protective equipment.

### Collection of new high-resolution internal exposure and effect data in the case studies

**Figure 4.**
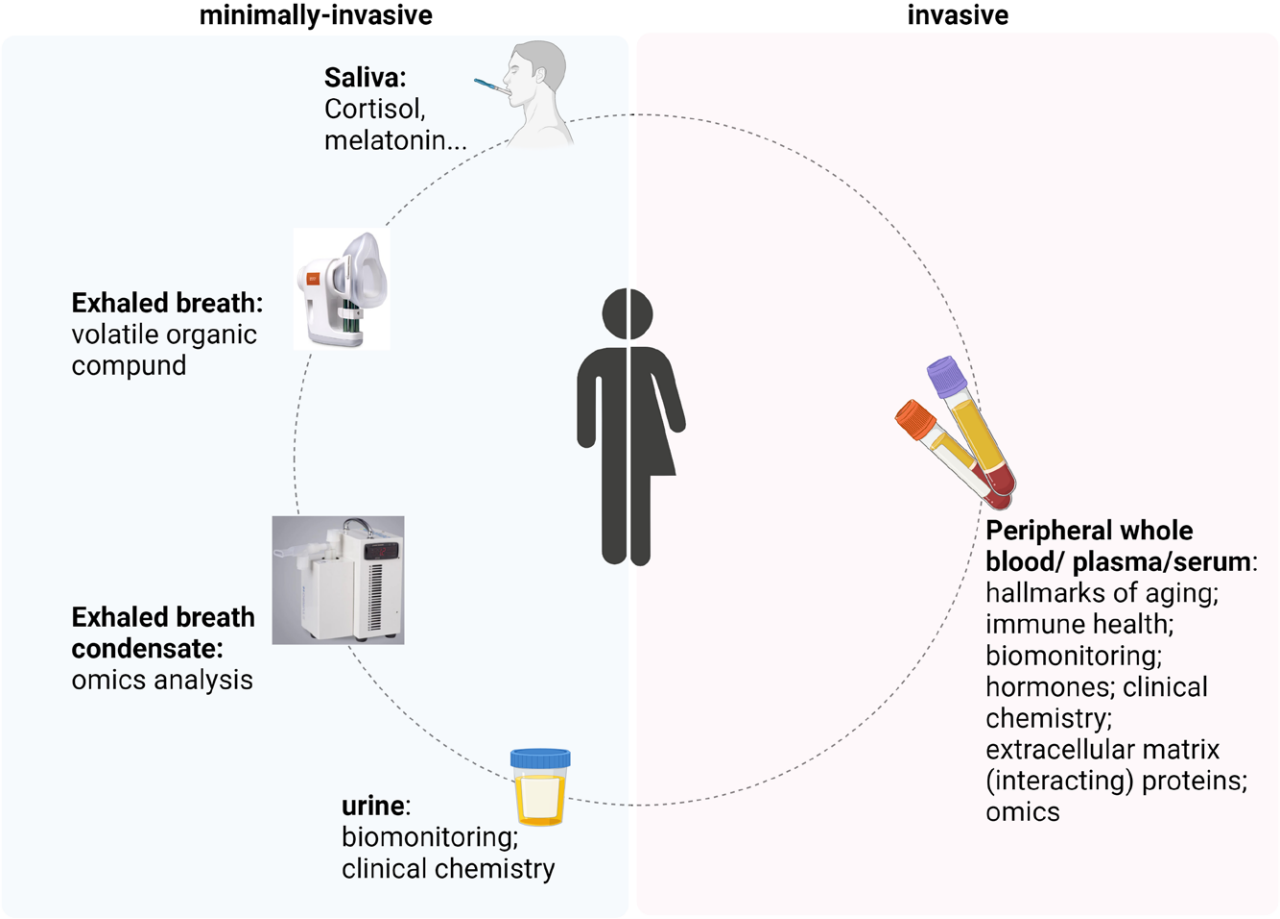
Overview of minimally invasive and invasive sampling and analyses in EPHOR case studies.

The internal exposome characterizes exposure biomarkers and biological pathways to link external exposure and health effects. In addition to blood collection using phlebotomy, minimally invasive biomatrices such as saliva, exhaled breath (EB) condensate, and urine will also be collected for analyses of several biomarkers. In addition, finger stick blood samples will be collected and stored as dried blood spot for analysis. For both case studies, samples will be collected during approximately one week, with exact timing and frequency of sample collection depending on the case study, the study center, and the type of sample. Self-collection of samples has been increased in the protocols due to coronavirus disease 2019 (COVID-19). Since self-collection will facilitate taking biological samples in occupational settings without impacting workers or working operations, this may also enhance opportunities after the COVID-19 pandemic. Figure 4 shows analyses foreseen in the case studies. Samples from case studies will be analyzed in a tiered approach, in which those individuals for whom the largest external exposure contrast or biomonitoring responses are discovered are prioritized for analysis of more markers. It is expected that a combination of conventional biomonitoring, single biomarkers of exposure/early effect and omics analyses as described below provides insight into the internal occupational exposome, as similar approaches have been successfully applied in other human cohort studies.

#### Markers in blood (invasive biomaterial sampling)

Blood will be collected via phlebotomy. Several analyses are foreseen in the protocols: hallmarks of aging (telomere length; mtDNA copy number), immune health (chitinase-3-like protein 1, clara cell secretory protein, interleukins, immunoglobulins), biomonitoring for chemicals, hormones (melatonin; corticosteroids, sex hormones), clinical chemistry, extracellular matrix proteins, and omics (epigenomics, proteomics, quantitative polymerase chain reaction [qPCR] of selected genes).

#### Markers obtained via minimally invasive biomaterial sampling

Forseen analyses include cortisol awakening response/diurnal cortisol slope in saliva and clinical chemistry, metals, and 1-hydroxypyrene in urine. EB will be explored as a novel noninvasive biological matrix. Owlstone ReCIVA Breath Biopsy technology will be used for collection. EB will be analyzed for omics similar as described for blood, to address interorgan similarities in biological pathways to discover minimally invasive biomarkers. EB also contains volatile endogenous and exogenous organic compounds, representing additional candidate biomarkers of exposure and/or effect. VOCs will be analyzed using gas chromatography/mass spectrometry. The analysis of exogeneous exhaled chemicals and/or endogeneous biological markers in exhaled breath to characterize exposure or early biological responses has been successfully applied in subway workers, firefighters, welders, metal and machining industries, airport workers, ceramics production facilities, and hospital cleaners.^48–60^

### Methods for storage and interpretation of working life exposome data

#### Data storage and sharing

The human exposome data structure is a high-dimensional collection of highly heterogeneous exposure variables. Given the scale needed in exposome studies, data will be provided by many institutes. For data management, the Yoda platform developed by Utrecht University redundant will be used as a findable, accessible, interoperable, and reusable (FAIR) data point for large amounts of research data during all stages of EPHOR. Yoda facilitates collaboration on publication of long-term archiving of and referring to data. Newly collected data along with their metadata are shared via internet within a closed authorized user group. Research data integrity is enhanced through the use of the Yoda Vault, in which data become read-only.

#### Exposure-response analyses

Occupational settings often involve more than one exposure of interest. As the underlying principle behind the exposome is the assessment of many different exposures, we will compile and compare methods capable of handling multiple exposures in exposure-response analyses, including variable selection methods (e.g., penalized regression). Additional methods will be explored, either identifying groupings in a data-driven way or by forcing logical groupings of exposures,^61^ such as by chemical class using a battery of latent class and clustering approaches. For correctly handling complex exposure-time-response (ETR) relationships, compartmental (multistate) models, (two-stage) clonal expansion models, and exposure rate models have been successfully applied, but it is not yet clear how they compare in terms of data requirements and inference. We will compile these methods, apply, and compare them in the EPHOR Mega Cohort. In parallel, we will investigate to what extent existing ETR models can be modified to allow for multiple exposures and nonlinear exposure-response relations and to incorporate biological data to strengthen these models (effect markers, omics).^62^ Hierarchical regression will be used as a general approach, including Semi-Bayes adjustment, that aims at improving the validity of standard maximum-likelihood estimates in the presence of multiple comparisons by incorporating similarities between the exposures of interest in a second-stage model.^63^

#### Biological pathways

Internal exposure and effect data (omics, biomarkers) will be subjected to pathway analyses to understand relations between external exposure and health outcome. The first level of analysis involves qualitative pathway analysis to retrieve sets of genes, the expression of which has changed due to exposure. This analysis will include combinations of different omics data sets and also compare exhaled breath and blood omics to develop noninvasive biomarkers. Gene set enrichment analysis to interpret omics data from occupationally exposed individuals has been applied successfully before.^64–67^ The second level of bioinformatics involves quantitative pathway-based exposure to health effect modeling. Existing scientific knowledge, exposure, and pathway content will be retrieved from text mining of scientific literature and toxicological databases, to construct qualitative exposure to adverse outcome pathway networks. Using these, causal modeling (e.g., reverse causal reasoning, physiologically based kinetic modeling) will be performed with omics, biomonitoring, and biomarker data from case studies to quantitatively predict health in relation to exposure.

In toxicology, pathway knowledge retrieved via textmining has been successfully combined with omics data, to analyze for the enrichment of genesets to identify similarities in chemical exposures.^68^ However, quantitative modeling using pathway networks to link exposure to health outcomes, also referred to as systems epidemiology,^69^ is novel in occupational epidemiology.

### Ethical considerations

The approach adopted in EPHOR for the advancement of occupational health gives rise to ethical and legal challenges regarding in particular issues of consent, privacy, and transborder flow of health-related data. For the large-scale pooling of existing cohorts from different countries, data transfer, especially across borders, has proven difficult due to legal and ethical challenges. This is addressed through the use of DataSHIELD software, which secures the privacy-preserving analysis of data that remain at the institute that owns the data. Data access agreements are being realized in compliance with the general data protection regulation and the national regulatory frameworks of participating countries. Collection of new data for the case studies will be undertaken in multiple study centers spread over different countries. Data and material flow overviews have been created, and data and material transfer agreements are underway in order to facilitate central data analyses.

#### Health impact assessment

Health impact assessment (HIA) is important for translating scientific evidence into preventive actions by policy makers or occupational health practitioners as it predicts the future health consequences of interventions such as policies or new procedures. Within EPHOR, we will develop a conceptual framework for HIA based on the exposome principles, in contrast to HIA based on single exposure-health outcome pairs, as is currently the norm. The global burden of disease model^70^ will be used as the basis for transforming the single exposure, single-outcome method into a complex, multifactorial exposure-outcome approach, taking into account correlations and interactions and incorporating information about differential exposures and susceptibilities for vulnerable populations, where available. For the incorporation of work-specific health impact metrics, including working life expectancy and working years lost, we will build on previous work.^71^ For evaluating the applicability of the new HIA models to policy makers and occupational health practitioners, a set of hypothetical intervention scenarios will be developed and applied in a simulated cohort with input parameters based on the EPHOR Mega Cohort.

#### Working life exposome toolbox and stakeholder involvement

The knowledge and tools developed in the EPHOR project will be tailored to three groups of stakeholders. For scientists, methods and tools will be made available to expand the current knowledge base on the working life exposome in relation to health. These methods and tools include inventories, protocols, proof of concepts, decision support, and visualization tools and tutorials. For policy makers, data and tools for analyzing and assessing the impact of the working life exposome will be produced to support development of evidence-based and cost-effective preventive policies. For occupational health practitioners, data and tools for developing evidence-based and cost-effective preventive actions will be made available. All groups of stakeholders will be consulted during the development of all aspects of the toolbox, to take into account the values, needs, and expectations of these stakeholders during development.

## Strengths and limitations

EPHOR will be the first large study worldwide to deliver knowledge and methods to characterize the working life exposome in relation to NCDs. To maximize the research yield, EPHOR’s study approach involves the unique combination of analyses of large-scale pooling of existing cohorts, with two exposome case studies involving broad implementation of new exposome technologies. An important strength is the use of the large body of occupational cohort studies in Europe. Europe has a long tradition of occupational health research and currently has some of the most valuable registry-, population-, and industry-based cohorts worldwide. These existing cohorts are an invaluable resource since they have collected a wealth of data on lifetime occupational histories in addition to more general characteristics of the population.

Pooling data from many cohorts is challenging, however. This involves getting permissions for accessing the cohorts and JEMs, for aligning and agreeing upon metadata descriptors and conducting data harmonization. Subsequent data integration poses challenges involved in the analysis of distributed personal data within the boundaries of the General Data Protection Regulation. It also should be acknowledged that pooling cohort data in large quantities will come at a cost with respect to the quality of the data due to data harmonization at the level of the lowest common denominator leading to loss of information. However, previous endeavors pooling existing cohort studies across European countries have been shown to be fruitful. For example, the Nordic Occupational Cancer Study (NOCCA), a large follow-up study of 15 million working-aged persons derived from different cohorts in five Nordic countries and 2.8 million cancer cases diagnosed between 1961–2005.^72^ NOCCA has described risks of 84 cancer types in 54 occupational categories and occupational exposure to 30 documented and potential carcinogens. The estimated dose-response associations between exposures and cancers have been both novel findings as well as confirmations (or not) of findings from earlier smaller studies. To minimize exposure misclassification, a Nordic Job-Exposure Matrix (NOCCA-JEM) for 30 documented and potential carcinogens, including asbestos, formaldehyde, wood dust, quartz, and several specific metals and organic solvents was developed,^30^ similar to the intended EuroJEM within EPHOR. The order of magnitude of the NOCCA study, which is mainly based on register-based data, will not easily be surpassed by adding industrial and nonregister population-based cohorts. However, data richness may be improved as national registers typically lack the in-depth occupational histories and lifestyle and behavioral data obtained from questionnaires or physiological data obtained from detailed clinical examinations and are likely to offer less diversity with respect to the social and cultural environment. Another example is the EU Child Cohort network which has recently developed a FAIR data resource consisting of (a protocol for) harmonized core variables and data catalogue for over 17 birth cohorts within the EU LifeCycle project.^73^ The advantages of creating a large data set despite the loss of information as described by this consortium are exploring multiple interactions, complex relationships, health effects with a small risk, rare diseases, and sub populations^74^ and correspond largely to the specific objectives of the EPHOR Mega Cohort approach.

Studying the (working life) exposome is complicated, as nongenetic factors are numerous, partly undefined, and vary greatly between people and over time. Technological advances, such as high-resolution mass spectrometry, sensor development, and artificial intelligence, have previously been used as first steps toward new data collection for a more comprehensive assessment of the exposome.^16,37,38,74^ EPHOR’s case studies will build on the methodical developments and experiences from earlier exposome studies, which have focused on environmental health or vulnerable groups like children.

A challenge with respect to new data collection is to find the right balance between taking a holistic angle and sample size. In the case studies, we have included an appropriate number of individuals to be enrolled in terms of power for some key outcomes. However, as many exposome technologies are used, some of which are still under development, and data distributions in a normal population are only partially known, sufficient numbers of data points cannot be guaranteed for all parameters. We, therefore, use a combination of targeted and agnostic potential risk factors and internal markers. Also, we have focused the case studies on either one exposure situation or one health outcome to increase power. Also, for the internal exposome, a tiered approach will be followed, enhancing the chances of pathway and biomarker discovery.

Participation in the European Human Exposome Network (EHEN) will enable exchange of experience with similar methods and technologies with concurrent EU exposome projects. EPHOR will contribute uniquely to EHEN by characterizing the working life exposome as an essential factor in the development of NCDs. Also, the focus on occupational exposure settings, with well-defined populations and exposure levels that can be higher and more frequent for e.g., chemicals and noise^75^ and that can generally be well characterized, may provide a unique setting for developing and demonstrating exposome methods.

## Conclusion

**Figure 5.**
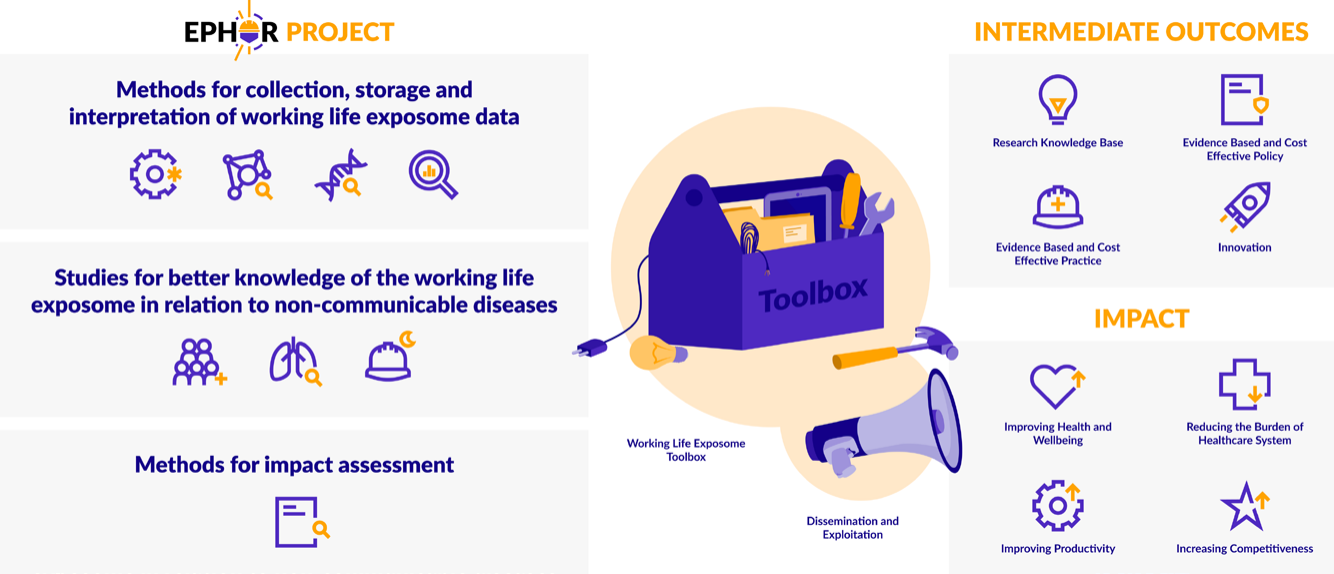
Overview of EPHOR and its expected intermediate outcomes and impact.

EPHOR is the first large study that applies the exposome concept to working life health. EPHOR will lay the groundwork for identifying risks from (un)known and interacting exposures, including nonoccupational exposures, during working life spanning vulnerable life stages (e.g., young adult life, the reproductive period, and aging working life). The risk estimates, methods, and tools will be made available in a toolbox, which enables researchers, policy makers, and occupational health practitioners to continuously include new knowledge in the policy making and industrial hygiene process. This facilitates the development of improved risk mitigating and disease preventive measures, resulting in a more resilient population at higher age and with reduced health care costs. Ultimately, EPHOR aims to contribute to reducing the burden of NCDs on the EU health care systems, to improving the health and well-being of the EU population and productivity of the EU workforce and to increasing the competitiveness of EU industry (Figure 5). Ensuring a safe and healthy work environment for over 170 million workers is a strategic goal for the European Commission.^8^

## ACKNOWLEDGMENTS

The Exposome Project for Health and Occupational Research (EPHOR) is funded by the European Union’s Horizon 2020 research and innovation programme under grant agreement number 874703. We would like to acknowledge Dr. Debbie Jarvis and Dr. Cecilie Svanes for their leading and coordinating role in European Community Respiratory Health Survey. M.C.T. is funded by a Ramón y Cajal fellowship (RYC-2017-01892) from the Spanish Ministry of Science, Innovation and Universities and cofunded by the European Social Fund. ISGlobal acknowledges support from the Spanish Ministry of Science and Innovation through the “Centro de Excelencia Severo Ochoa 2019-2023” Program (CEX2018-000806-S) and support from the Generalitat de Catalunya through the CERCA Program. The authors would like to thank Prof. John W. Cherrie for his inspiring contributions to the initial conception of the EPHOR project.

## Supplementary Material


